# Tetra-fluorinated aromatic azide for highly efficient bioconjugation in living cells†

**DOI:** 10.1039/c8ra09303b

**Published:** 2018-12-19

**Authors:** Xuekang Cai, Dan Wang, Yasi Gao, Long Yi, Xing Yang, Zhen Xi

**Affiliations:** Department of Nuclear Medicine, Peking University First Hospital Beijing 100034 China yangxing2017@bjmu.edu.cn; State Key Laboratory of Organic–Inorganic Composites, Beijing University of Chemical Technology 15 Beisanhuan East Road, Chaoyang District Beijing 100029 China; State Key Laboratory of Elemento-Organic Chemistry, Department of Chemical Biology, National Pesticide Engineering Research Center (Tianjin), Nankai University Tianjin 300071 China zhenxi@nankai.edu.cn +86 22 23504782; Collaborative Innovation Center of Chemical Science and Engineering (Tianjin) China; Peking University School of Medical Technology Beijing 100191 China

## Abstract

We developed a fast strain-promoted azide–alkyne cycloaddition reaction (SPAAC) by tetra-fluorinated aromatic azide with a kinetic constant of 3.60 M^−1^ s^−1^, which is among the fastest SPAAC ligations reported so far. We successfully employed the reaction for covalent labelling of proteins with high efficiency and for bioimaging of mitochondria in living cells. The reaction could be a generally useful toolbox for chemical biology and biomaterials.

## Abstract 2

A fast strain-promoted azide–alkyne cycloaddition based on tetra-fluorinated aromatic azide was developed and applied to label proteins and living cells with high efficiency.

## Graphical abstract

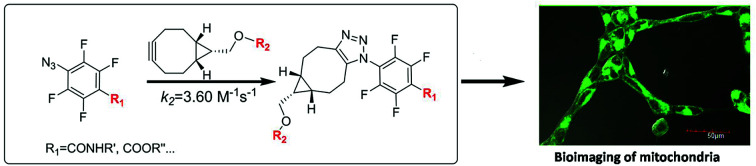

Strain-promoted azide–alkyne cycloaddition (SPAAC) as an efficient bioconjugation tool has recently found a wide range of applications in chemical biology, materials science and medical science.^1–3^ In 2001, Sharpless *et al.*, first introduced the concept of click chemistry that emphasized on the rapid synthesis of useful new compounds and combinatorial libraries through heteroatom links (C–X–C).^4^ To overcome the limitations for classical copper-catalyzed azide–alkyne cycloaddition (CuAAC),^5^ Bertozzi improved the method using strained cyclooctynes to react with azide in 2004, which effectively avoids the transition metal catalysts (such as Cu) and makes it more general for bioconjugations.^6,7^ It gave birth to the development of SPAAC. But one of the limitations is the relative low reaction rate compared with other biorthogonal reactions and a high rate could be beneficial for labelling low abundance or transient structures with short-lived signal molecules, such as radioisotopes for imaging.^8^ During the past decade, efforts have been made to further improve the method. Structure modified cyclooctynes, including aliphatic cyclooctynes^9^ and (di)benzoannulated cyclooctynes^10^ have been investigated to accelerate the reaction, and recently the modified azides was also reported to remarkably increase SPAAC rate.^11^

We have been interested in the development of fast chemoselective reactions for bioconjugations.^12^ We reported *o*,*o*′-difluorinated aromatic azide can accelerate both the reaction rate significantly on SPAAC and H_2_S-mediated reduction of the azide.^12*c*^ Therefore, we envision that multi-fluorinated aromatic azide may have interesting properties for fast and convenient bifunctional conjugation. 4-Azido-2,3,5,6-tetrafluorobenzoic acid (1) was selected for investigation. Here, we report the SPAAC kinetic properties of 1 and its applications for protein labelling *in vitro* and for bioimaging of subcellular organelles in living cells (Fig. 1).

**Fig. 1:**
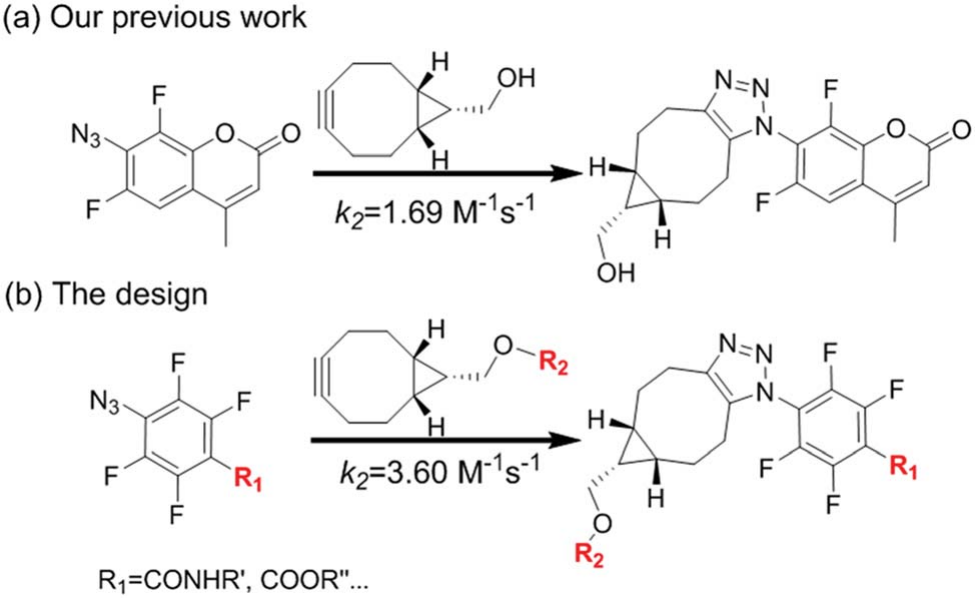
Rational design of SPAAC reaction based on multi-fluorinated aromatic azide. (a) SPAAC reaction based on *o*,*o*′-difluorinated aromatic azide reported in our previous work; (b) SPAAC reaction based on tetra-fluorinated aromatic azide for faster and bifunctional ligation.

To test our idea, the reaction between 1 and [1*R*,8*R*,9*S*]-bicyclo[6.1.0]non-4-yn-9-yl methanol (2) was studied for SPAAC reaction, monitored with time-dependent ^1^H NMR. To our delight, the reaction could finish within 5 min. As shown in Fig. 2, the ^1^H-NMR signal of 2 (40 mM) completely changed to the product (3) after adding 1 for 5 min, and there's no further change at 15 min and 2 hours in NMR spectra. The resulted product was also confirmed by high resolution mass spectrum. This result implies that the 4-azido-2,3,5,6-tetrafluorobenzoic acid derivatives can be used for this fast SPAAC reaction. But NMR is limited by its relative low sensitivity for accurate testing the kinetic rate at micromolar concentration.

**Fig. 2:**
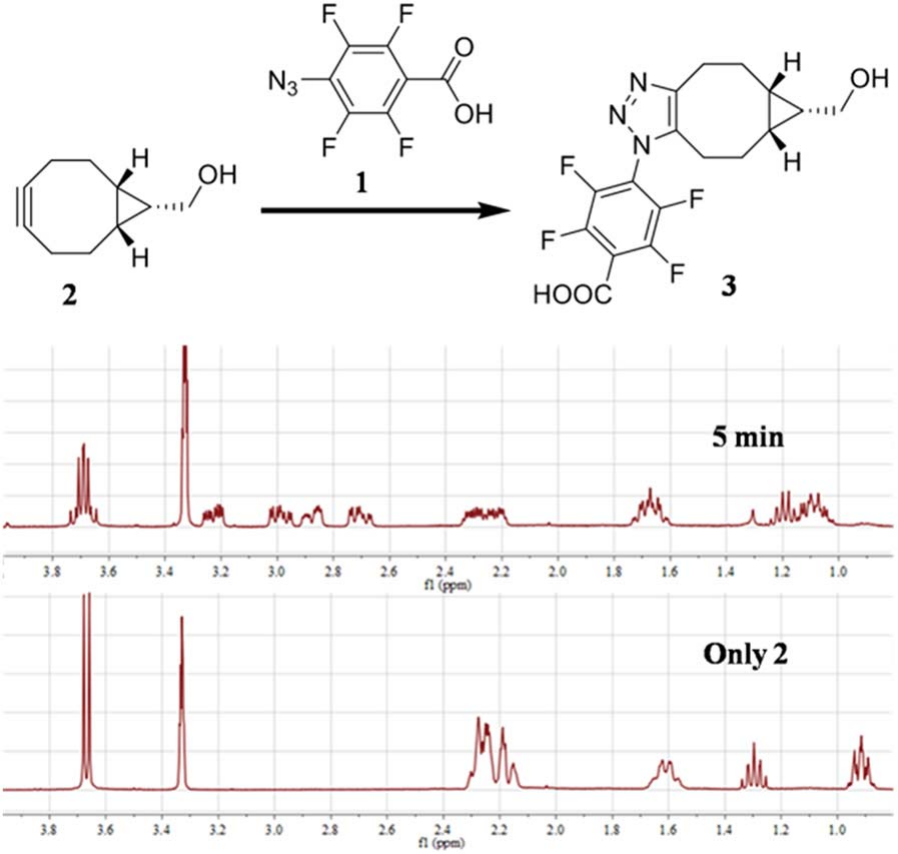
^1^H NMR analysis of the reaction between 1 (120 mM) and 2 (40 mM). The reaction was carried out in CD_3_OD.

In order to quantify the kinetic rate, we designed a procedure based on fluorescence resonance energy transfer (FRET) method to monitor this fast SPAAC reaction. We synthesized the azide compound 4 conjugated to an azo-quencher and the cyclooctyne compound 5 conjugated to a BODIPY-dye (Fig. 3). All the compounds were isolated and characterized by NMR and HRMS. After the reaction between 4 and 5, 6 could formed in which BODIPY fluorescent signal was quenched due to the FRET effect. Such fluorescence change could be employed to monitor the reaction in a real-time.

**Fig. 3:**
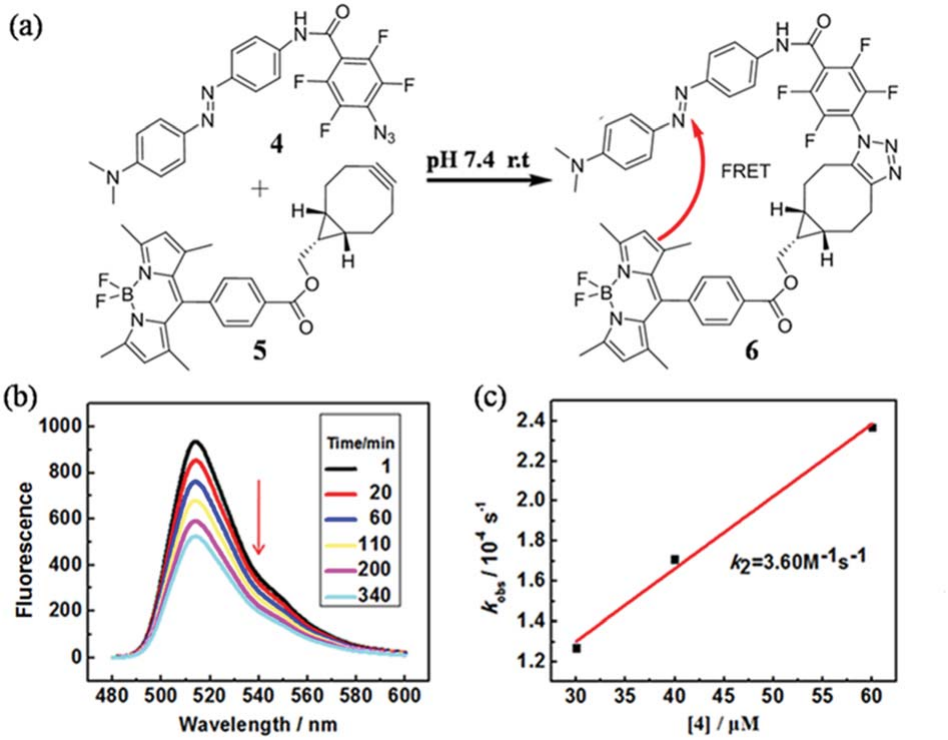
(a) Chemical structures of 4 and 5 and their SPAAC reaction to produce 6. (b) Time-dependent fluorescence spectra of 5 (2 μM) upon treated with 4 (30 μM) in PBS (50 mM, pH 7.4, containing 70% CH_3_CN) at room temperature (excitation, 473 nm). The reaction time is shown inset. (c) The linear relationship between the concentration of 4 and *k*_obs_. The slope of the best linear fitting gives the reaction rate *k*_2_ (M^−1^ s^−1^).

The reaction between 4 and 5 was set up in PBS buffer (2 μM 5, 50 mM, pH 7.4) and the maximum emission at 511 nm was monitored with the excitation at 473 nm. The pseudo-first-order rate *k*_obs_ was determined by fitting the data with a single exponential function. The linear fitting between *k*_obs_ and the concentrations of 4 gave the reaction rate (*k*_2_) as 3.60 M^−1^ s^−1^. It's two-fold faster than *o*,*o*′-difluorinated aromatic azide that we reported earlier, 1500-fold faster than the original SPAAC reaction and among the fastest SPAAC reactions reported so far.

With this highly efficient reaction, covalent protein labelling was first tested as its application. *N*-Hydroxysuccinimide ester of 4-azido-2,3,5,6-tetrafluorobenzoic acid was synthesized as bifunctional labelling compound (7) for amide and SPAAC reactions. We chose bovine serum albumin (BSA) and lysozyme as model proteins considering their different sizes and functions.^8^ As shown in Fig. 4a, we tested to label protein first with 7 and then conjugated a dye to the protein using fluorescent cyclooctyne (5). BSA or lysozyme was treated with 0.5 mM 7 in PBS buffer (50 mM, pH 8.5, containing 10% DMSO) for 2 h to incorporate tetra-fluorinated aromatic azide into the protein. After removing of small molecules, the azide labelled protein was incubated with 1 mM 5 for another 2 h to achieve SPAAC protein fluorescent dye labelling. As a control, the azide labelled protein was also treated with Na_2_S for 10 minutes to reduce azide into amine before incubating with 5, so to prove the reaction specificity. The labelled proteins were analysed with SDS-PAGE either stained by Coomassie blue or excited under UV lamp to visualize the desired protein. The results are shown in Fig. 4b. The strong fluorescent labelled BSA (lane 1) and lysozyme (lane 4) could be observed after the reaction with tandem addition of 7 and 5, while the controls of 5 only (lane 2 and 5) and Na_2_S treated labelling (lane 3 and 6) did not show any fluorescent signal. The results indicated that tetra-fluorinated aromatic azide-cyclooctyne is a highly efficient SPAAC reaction for protein labelling.

**Fig. 4:**
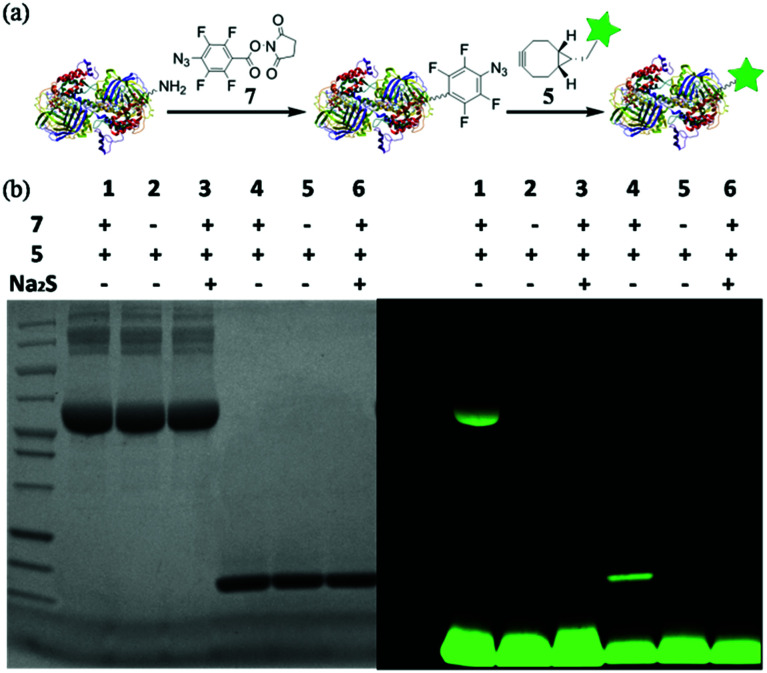
Fluorescence labeling of BSA and lysozyme *via* the SPAAC reaction. (a) The fluorescence labelling strategy. (b) 15% SDS-PAGE of BSA (lane 1–3) and lysozyme (lane 4–6) was imaged under UV lamp (right) and then stained by Coomassie blue (left).

To future explore the biocompatibility of this SPAAC reaction in living cells, we tested it for bioimaging of the subcellular organelle mitochondria. Triphenylphosphines are known to enrich into mitochondria mainly due to its positive charged character.^13^ We designed and synthesized compound 8, with the azide conjugated to a triphenylphosphine. Upon incubating 8 with the cells, we expected it would target mitochondria, so we can use SPAAC reaction to label the cells with fluorescent compound 5 (Fig. 5a). Human embryonic kidney cells 293 (HEK 293) were chosen for the experiment. The cells were first treated with 8 (10 μM) for 20 min to accumulate tetra-fluorinated aromatic azide into mitochondria, and then incubated with 5 μM 5 for another 30 min. After the non-specific fluorescent signal was washed away, the cells were imaged under the excitation of 488 nm. The experimental conditions and results were shown in Fig. 5b. The cells could only be fluorescence-labelled when treated with both 8 and 5, and the imaging signal all localized on mitochondria. The result proved both the feasibility and biocompatibility for the application of this improved SPAAC conjugation at cellular level. Its superior kinetic character (*k*_2_ of 3.60 M^−1^ s^−1^) may even enable *in vivo* applications for pretargeted imaging,^14^ which is currently under investigation.

**Fig. 5:**
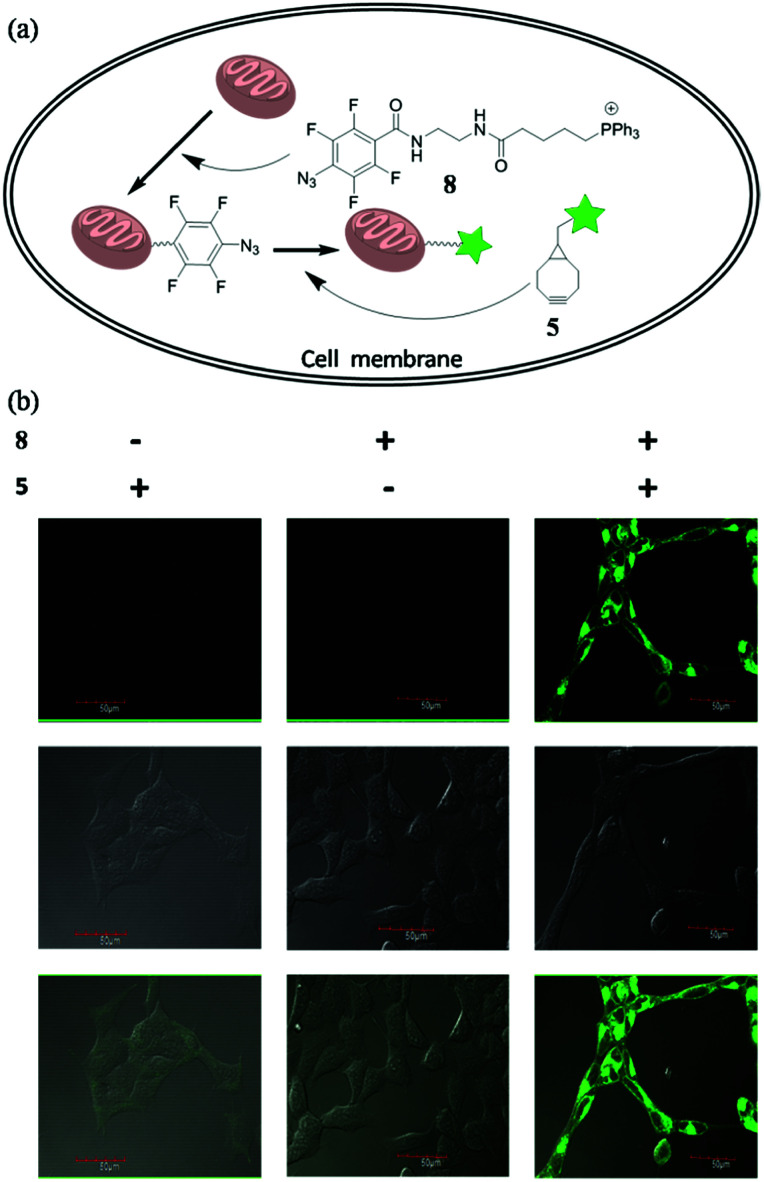
Fluorescence labelling of mitochondria in living cells *via* the SPAAC reaction. (a) The fluorescence labelling strategy. (b) Fluorescence images of HEK293 cells. HEK293 cells were treated for 30 min only with 10 μM of 5 (left); 20 min only with 10 μM of 8 (middle); 20 min with 10 μM of 8 followed by 5 μM of 5 for 30 min (right).

## Conclusions

In summary, we investigated tetra-fluorinated aromatic azide for SPAAC reactions with cyclooctyne. The reaction rate could reach 3.60 M^−1^ s^−1^ for 4 and 5, which is among the fastest SPAAC reactions reported so far. The commercially available agent 4-azido-2,3,5,6-tetrafluorobenzoic acid (1) enables its convenient application as bifunctional biorthogonal tool. We have successfully employed the reaction for covalent labelling of proteins and the mitochondria in living cell. *In vivo* applications are currently under investigation.

## Conflicts of interest

There are no conflicts to declare.

## Supplementary Material

RA-009-C8RA09303B-s001
