# Supplementary material for: Tetra-fluorinated aromatic azide for highly efficient bioconjugation in living cells
Source: RSC Adv. 2018 Dec 19;9(1):23–6. doi: 10.1039/c8ra09303b (PMC9059488; doi:10.1039/c8ra09303b)
Supplement: RA-009-C8RA09303B-s001 [file RA-009-C8RA09303B-s001.pdf]

Electronic Supplementary Material (ESI) for RSC Advances.
This journal is © The Royal Society of Chemistry 2018

Supporting Information

Tetra-fluorinated Aromatic Azide for Highly Efficient Bioconjugation Ligation in living cells†

Xuekang Cai,‡[a,b] DanWang,‡[c] Yasi Gao,[b] Long Yi,[b,d] Xing Yang*[a,e] and Zhen Xi*[c,d]

Table of Contents

1. **Model reaction** 2
2. **Reaction kinetics of this SPAAC** 3
3. **Fluorescence labelling of BSA and lysozyme** 4
4. **Fluorescence labelling of mitochondria in living cells** 4
5. **Synthesis of probes** 4-6
6. **Supporting NMR and MS spectra** 7-15

1. Model reaction

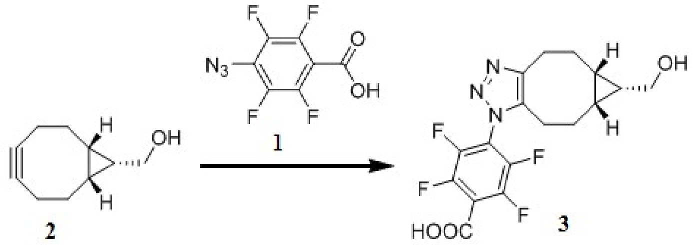

1 (39 mg, 0.17 mmol) and **2** (30 mg, 0.2 mmol) were dissolved in the mixture of MeOH and CH_3CN. The mixture was stirred at room temperature over night and protected from light. The solvent was evaporated under reduced pressure. Column chromatography (CH_2Cl_2/MeOH = 100/3) of the crude product over silica gel gave **3** (50 mg, 77%), R_f=0.43 (CH_2Cl_2/MeOH = 7/3). ^1H NMR (400 MHz, Methanol-d_4) δ 3.70 – 3.55 (m, 2H), 3.30 (s, 1H), 3.22 – 3.09 (m, 1H), 2.98 – 2.87 (m, 1H), 2.86 – 2.76 (m , 1H), 2.68 – 2.63 (m, 1H), 2.28 – 2.10 (m, 2H), 1.68 – 1.54 (m, 2H), 1.18 – 1.08 (m, 1H), 1.08 – 0.94 (m, 2H). ^{13}C NMR (101 MHz, Methanol-d_4) δ 161.0, 147.2, 146.2, 145.5, 144.7, 143.0, 138.6, 118.6, 117.7, 59.5, 26.7, 23.7, 23.2, 22.7, 22.0, 20.6, 20.5. ^{19}F NMR (377 MHz, Methanol-d_4) δ -140.5, -147.2.

2 (3 mg, 0.020 mmol) was dissolved in 0.5 mL CD_3OD, and then **1** (14.1 mg, 0.060 mmol) was added to the solution. The mixture was measured by ^1H NMR at 0min, 5 min, 15 min, 30 min, 1 h, 2 h (set 0 min as only **2**).

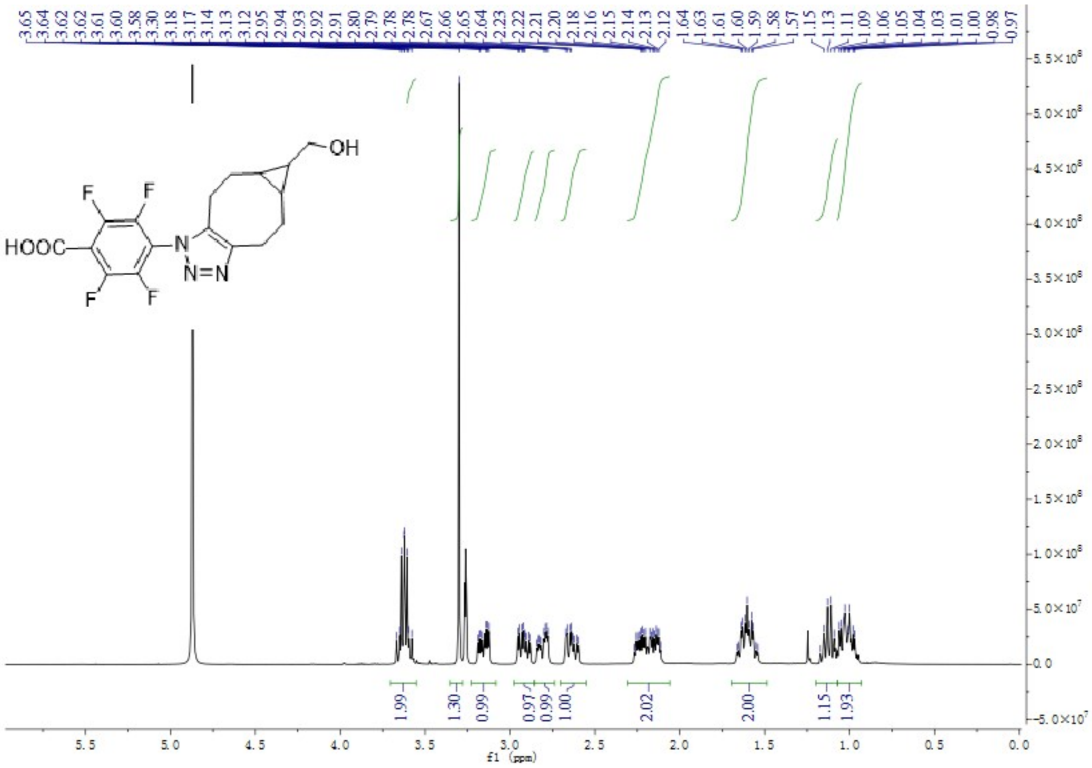

Fig. S1 ^{1}HNMR spectrum of the reaction product for **1** and **2**.

2. Reaction kinetics of this SPAAC

Fluorescent kinetics analysis: 2 μL **5** (2 mM) in DMSO and 2-12 μL **4** (10 mM) in DMSO was added to 2 mL PBS (50 mM, pH 7.4, containing 70% CH_3CN). The progress of the reaction was monitored by fluorescence spectrophotometry with excitation at 473 nm. The time-dependent emission at 511 nm was shown in Fig. S2. The pseudo-first-order rate, k_{obs} was determined by fitting the fluorescence intensity data with single exponential function. The linear fitting between k_{obs} and concentrations of **4** gives the reaction rate (k_2). The reaction product of **4** and **5** was confirmed by HRMS (Figure 3S).

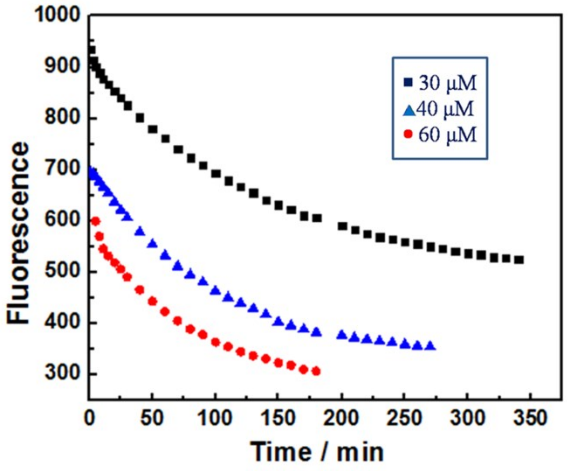

Fig. S2 The time-dependent fluorescence intensity at 511 nm of **5** (2 μM) in the presence of different concentrations of **4**.

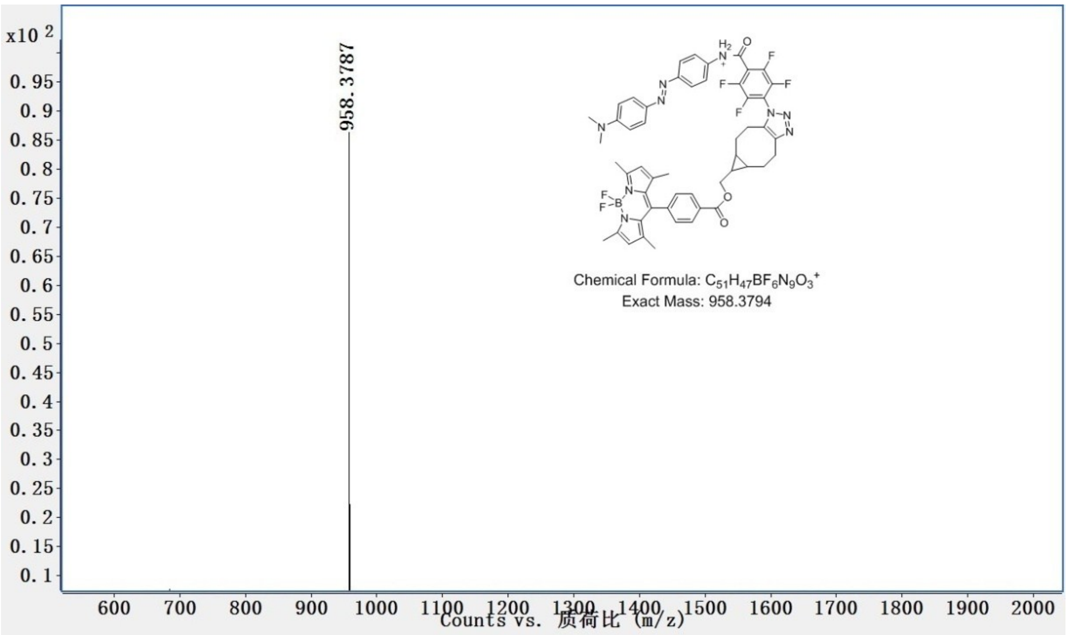

Fig. S3 High resolution mass spectrum of the reaction product for **4** and **5**.

3. Fluorescence labelling of BSA and lysozyme

100 μM BSA and lysozyme were treated with 0.5 mM **7** in 50 mM PBS (pH 8.5, contain 10% DMSO) for 2 h at room temperature respectively. Then the free **7** need to be removed by a desalting column (Zeba Spin Desalting Columns, 0.5 mL, Thermo scientific). **7**-labelled protein was placed in the center of the resin bed after equilibrating column. Then the sample was placed in new microcentrifuge collection tube and centrifuged at 1500 × g for two minutes. Subsequently, the **7**-labelled protein was incubated with 1 mM **5** for another 2 h. The control group was also treated with 2 mM Na_2S for 10 minutes at room temperature. The labeled protein suspension was mixed with loading buffer and directly used for electrophoresis in 15% SDS-PAGE. The gel was firstly imaged under UV lamp to detect whether the protein was fluorescently labelled. Then, the gel was stained by Coomassie brilliant blue and imaged.

4. Fluorescence labelling of mitochondria in living cells

Cell culture: HEK-293 cells were cultured at 37°C, CO_2 (5%) air environment in high glucose DMEM (GIBICO) supplemented with FBS (10%), penicillin (100 μg/ml), streptomycin (100 μg/ml) and l-glutamine (4 mm). The cells were maintained in exponential growth phase, and then seeded in a glass-bottom 35 mm plate (~ $2x10^4$ cells per well). Cells were passaged every 2–3 days and used between passages 3 and 10.
Confocal imaging experiments: Cells were imaged on an FV1000 inverted fluorescence confocal microscope (Olympus, Japan) with a UPLSAPO 40 x objective lens. All images were analyzed with Olympus FV1000-ASW software.
Co-localization: HEK 293 cells were incubated with **8** (10 μM) for 20 min and washed with 1mL PBS three times. Then the cells were incubated with **5** (5μM) for another 30 min and washed with PBS again. Finally, the medium was replaced by PBS, and cells were imaged *via* the green channels (lex= 488 nm, lem=500–600 nm). Control cells were treated with just **8** (10 μM) for 20 min.

5. Synthesis of probes

Synthesis of 3

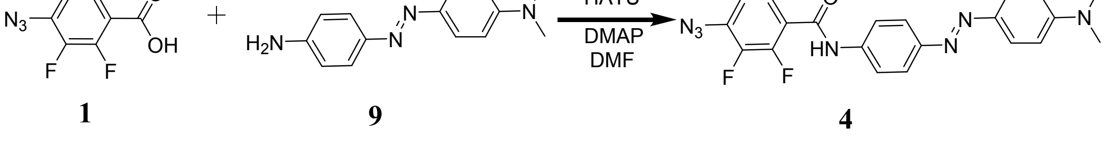

1 **9** **4**

4-Azido-2,3,5,6-tetrafluorobenzoic acid **1** (90.5 mg, 0.385 mmol) was dissolved in 1 mL DMF. Then HATU (199.6 mg, 0.525 mmol) and DMAP (64.14 mg, 0.525 mmol) were added to the solution. After stirring for 10 min, **9** (84.1 mg, 0.35 mmol) was added to the solution. The mixture was stirred at room temperature under nitrogen protection over night. Subsequently, the mixture

was extracted with ethyl acetate and washed by water and brine. The ethyl acetate solution was dried by anhydrous sodium sulfate and concentrated under reduced pressure. The resulting residuewas purified by silica gel column chromatography with CH_2Cl_2/MeOH = 1000/4 and orange solid **4** (68 mg, 43%) was obtained, R_f=0.6 (CH_2Cl_2/MeOH=100/4). ^{1}H NMR (400 MHz, DMSO-d_6) δ 11.20 (s, 1H), 7.83 (s, 6H), 7.78 (d, J = 9.0 Hz, 2H), 6.83 (d, J = 9.0 Hz, 2H), 3.06 (s, 6H).^{13}C NMR (101 MHz, DMSO-d_6) δ 155.4, 152.4, 149.0, 144.3, 142.6, 141.8, 141.3, 138.7, 139.1, 124.6, 122.8, 121.6, 120.0, 111.9, 111.6, 39.8. ^{19}F NMR (376 MHz, DMSO-d_6) δ -142.6 , -151.2. HRMS(ESI): m/z $[M+H]^+$ calcd. For $C_{21}H_{15}F_4N_7O$: 458.1347; found: 458.1347.

Synthesis of 4

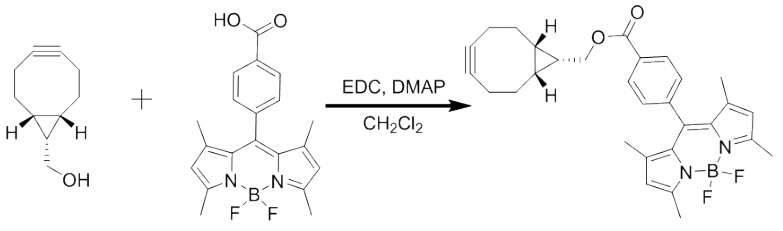

2 **10** **5**

2 (14.1 mg, 0.094 mmol) and **10** (27.2 mg, 0.074 mmol) were dissolved in CH_2Cl_2. Then 4-dimethylaminopyridine (24.3 mg, 0.200 mmol) was added. The mixture was stirred in ice bath and EDC (27.1 mg, 0.141 mmol) was added. After 30 min, the mixture was moved to room temperature and stirred for another 3 h. The solvent was removed under reduced pressure. Column chromatography (pure CH_2Cl_2) of the crude product over silica gel gave **5** (23.2 mg, 62.7%). ^{1}H NMR (400 MHz, $CDCl_3$) δ 8.21 – 8.15 (m, 2H), 7.43–7.37 (m, 2H), 5.99 (s, 2H), 4.46 (d, J = 8.2 Hz, 1H), 3.97 (s, 1H), 2.55 (s, 6H), 2.39 – 2.21 (m, 4H), 1.77 – 1.60 (m, 2H), 1.60 – 1.49 (m, 1H), 1.36 (d, J = 4.5 Hz, 6H), 1.32 – 1.16 (m, 2H), 1.11 – 1.00 (m, 1H), 0.98 – 0.79 (m, 1H). ^{13}C NMR (101 MHz, $CDCl_3$) δ 166.6, 166.2, 156.1, 143.0, 140.4, 140.0, 139.9, 131.2, 131.1, 130.9, 130.5, 128.5, 121.6, 98.9, 63.6, 52.5, 29.3, 21.6, 20.5, 17.6, 14.7, 14.6. HRMS (ESI): m/z $[M+H]^+$calcd. for $C_{30}H_{32}BF_2N_2O_2$: 501.2519; found: 501.2518.

Synthesis of 7

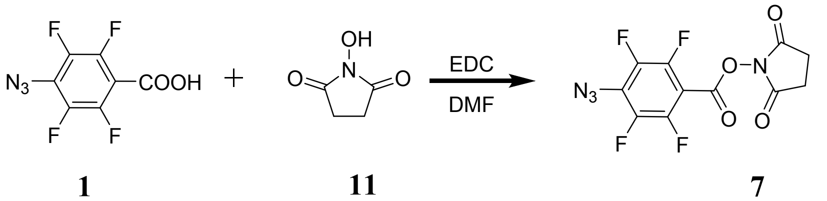

1 **11** **7**

4-Azido-2,3,5,6-tetrafluorobenzoic acid **1** (90.5 mg, 0.385 mmol) was dissolved in 1 mL DMF, then EDC (100 mg, 0.525 mmol) was added to the solution. After stirring for 10min, **11** (52.4 mg, 0.455 mmol) was added to the solution. The mixture was stirred at room temperature under

nitrogen protection for 3 h. Subsequently, the solvent was removed under reduced pressure. Column chromatography (pure CH_2Cl_2) of the crude product over silica gel gave **7** (66 mg, 47%), R_f=0.6 (CH_2Cl_2/MeOH = 100/2). ^{1}H NMR (400 MHz, $CDCl_3$) δ 2.91 (s, 4H). ^{13}C NMR (101 MHz, $CDCl_3$) δ 168.4, 155.3, 148.0, 145.2, 141.9, 139.3, 126.5, 102.2, 25.8. ^{19}F NMR (376 MHz, $CDCl_3$) δ -133.6, -149.8.

Synthesis of 8

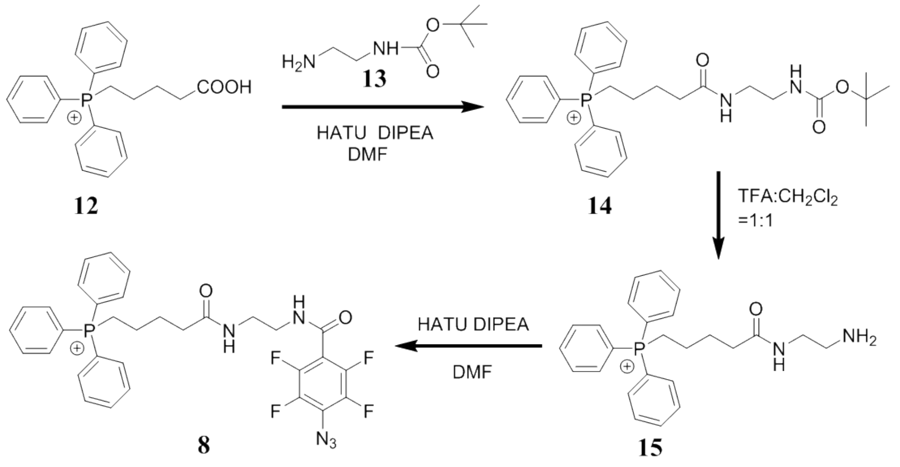

12 (860 mg, 2 mmol) was dissolved in 10 mL DMF, then HATU (913 mg, 2.4 mmol) and DIPEA (620 mg, 4.8 mmol) were added to the solution. After stirring for 10min, **13** (408 mg, 2.4 mmol) was added to the solution. The mixture was stirred at room temperature over night. Subsequently, the mixture was extracted with ethyl acetate (3×30 mL) and washed with water and brine. The ethyl acetate solution was dried by anhydrous sodium sulfate and concentrated under reduced pressure. Column chromatography (CH_2Cl_2/MeOH = 20/1) of the crude product over silica gel gave orange oil liquid **14** (1.05 g, 90%), R_f=0.3 (CH_2Cl_2/MeOH = 20/1). ^{1}H NMR (400MHz, Methanol-d_4) δ 7.91 (t, J = 7.0 Hz, 3H), 7.87 – 7.73 (m, 12H), 3.49 – 3.37 (m, 2H), 3.20 (t, J = 5.9 Hz, 2H), 3.10 (t, J= 6.1 Hz, 2H), 2.26 (t, J= 7.0 Hz, 2H), 1.87 (p, J = 7.1 Hz, 2H), 1.73 (dt, J = 15.2, 7.9 Hz, 2H), 1.44 (s, 9H).

14 (246 mg, 0.42 mmol) was dissolved in a mixture of 0.5 mL dichloromethane and 0.5 mL trifluoroacetic acid and the mixture reacted for 30 min. Then the solution was concentrated under reduced pressure and produced **15**. **15** was dissolved in 5 mL DMF, HATU (196 mg, 0.504 mmol) and DIPEA (130mg, 1.01 mmol) were added to the solution subsequently. After stirring for 10min, **1** (118 mg,0.504 mmol) was added to the solution and the mixture reacted for 4 h. 50 mL water was added to the solution to wash away DMF. The crude product was dissolved in methanol solvent and column chromatography (CH_2Cl_2/MeOH = 20/1) of the crude product over silica gel gave **8** (186 mg, 54%)**,** R_f=0.3 (CH_2Cl_2/MeOH = 100/6). ^{1}H NMR (400 MHz, Methanol-d_4) δ 7.91 (t, J = 7.2 Hz, 3H), 7.84 (t, J = 6.1 Hz, 4H), 7.79 (dd, J = 7.7, 3.9 Hz, 8H), 3.45 (q, J = 5.9 Hz, 4H), 3.38-3.35 (m, 2H)， 2.29 (t, J = 7.1 Hz, 2H), 1.89 (p, J = 7.3 Hz, 2H), 1.74 (h, J = 8.0 Hz , 2H). ^{13}C NMR (101 MHz, Methanol-d_4) δ 175.6, 160.4, 146.3, 143.8, 143.1, 140.6, 136.3(d, J = 2.9 Hz), 134.9, 134.8,

131.6, 131.5, 123.2, 120.3, 119.5, 113.2, 40.4, 39.9, 35.8, 27.6, 27.4, 23.0, 23.0, 22.9, 22.3. ^{19}F NMR (376 MHz, Methanol-d_4) δ -144.4, -153.4. ^{31}P NMR (162 MHz, Methanol-d_4) δ 23.7.

6. Supporting NMR and MS spectra

^{1}H-NMR

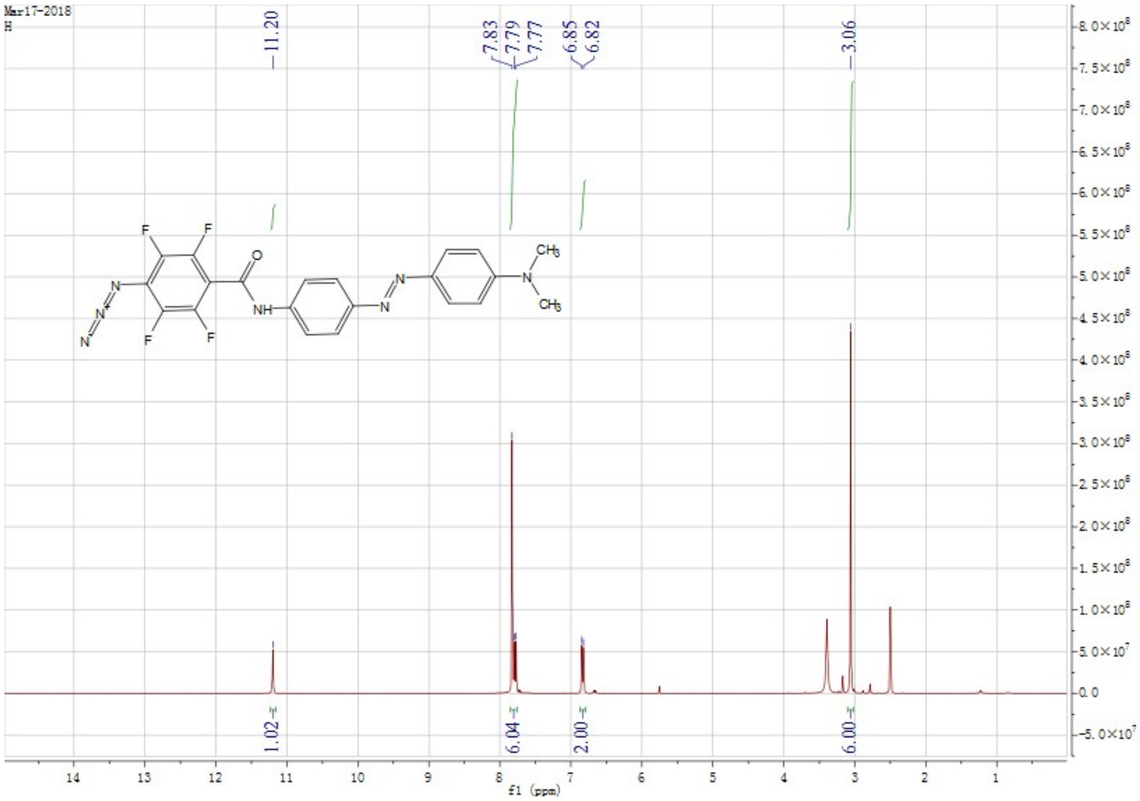

^{13}C-NMR

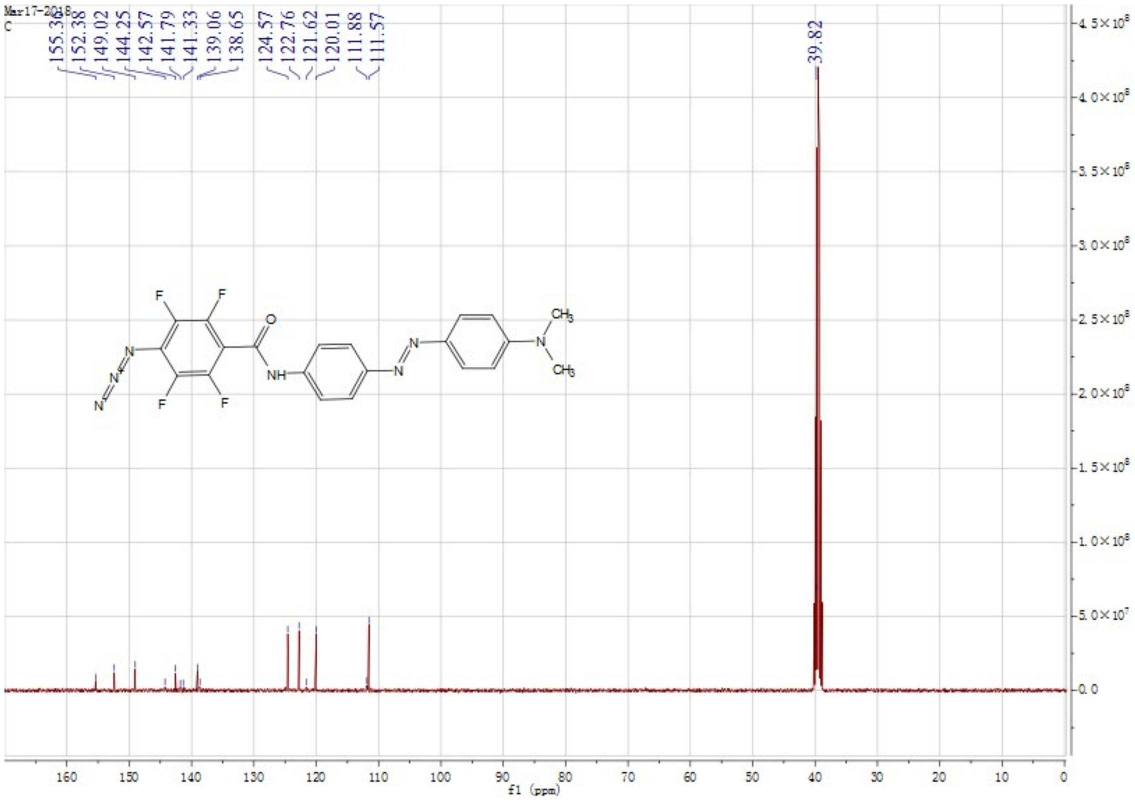
Mar17-2018
C
155.36
152.38
149.02
144.25
142.57
141.79
141.33
139.06
138.65
124.57
122.76
121.62
120.01
111.88
111.57
39.82
f1 (ppm)

^{19}F-NMR

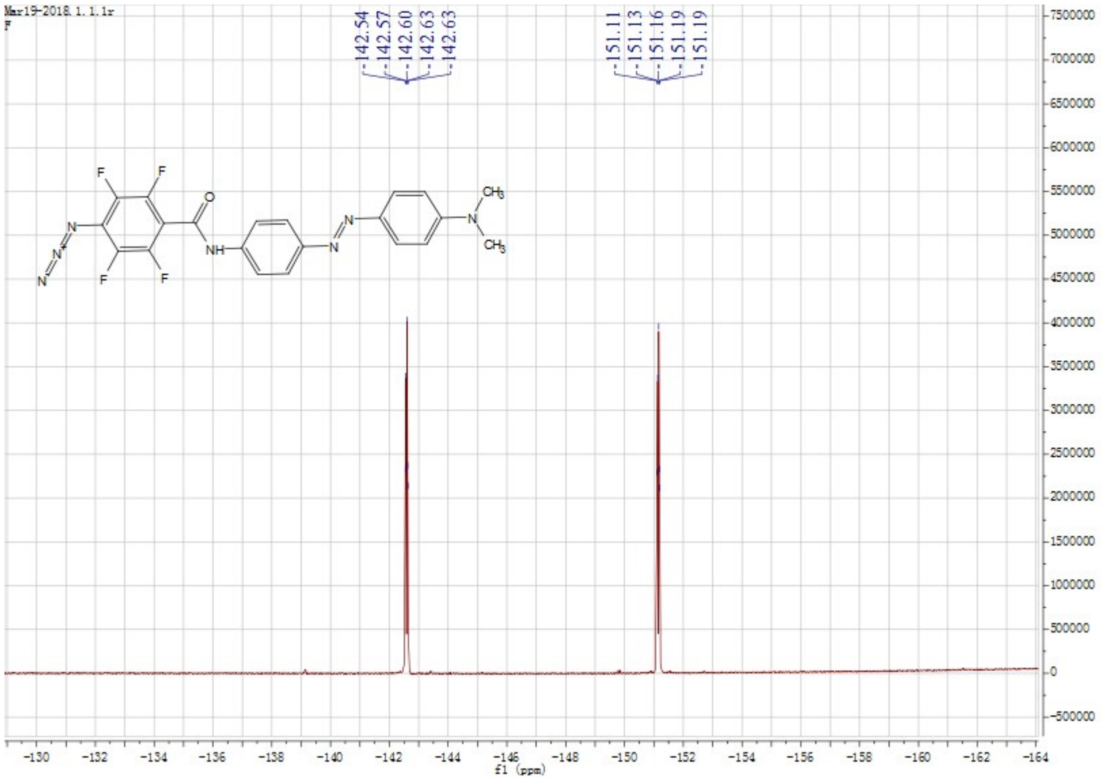
Mar19-2018.1.1.1r
F
-142.54
-142.57
-142.60
-142.63
-142.63
-151.11
-151.13
-151.16
-151.19
-151.19
f1 (ppm)

HRMS

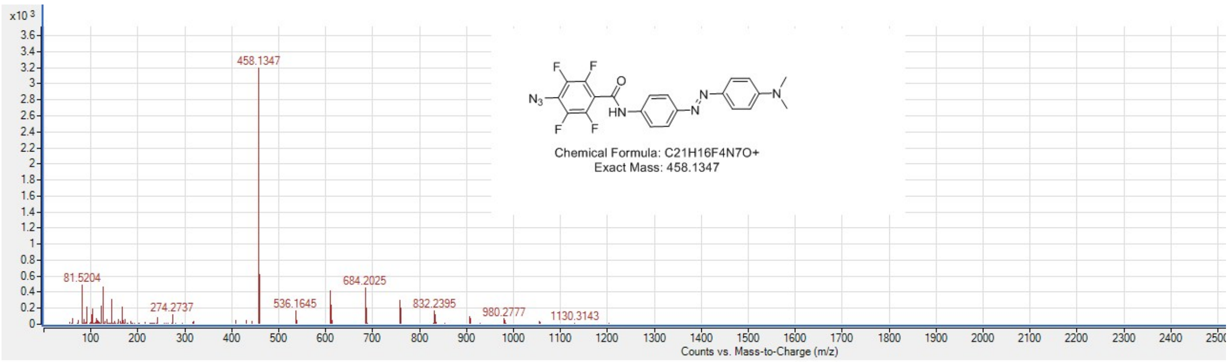
x10 3
458.1347
81.5204
274.2737
536.1645
684.2025
832.2395
980.2777
1130.3143
Chemical Formula: C21H16F4N7O+
Exact Mass: 458.1347
Counts vs. Mass-to-Charge (m/z)

^{1}H-NMR

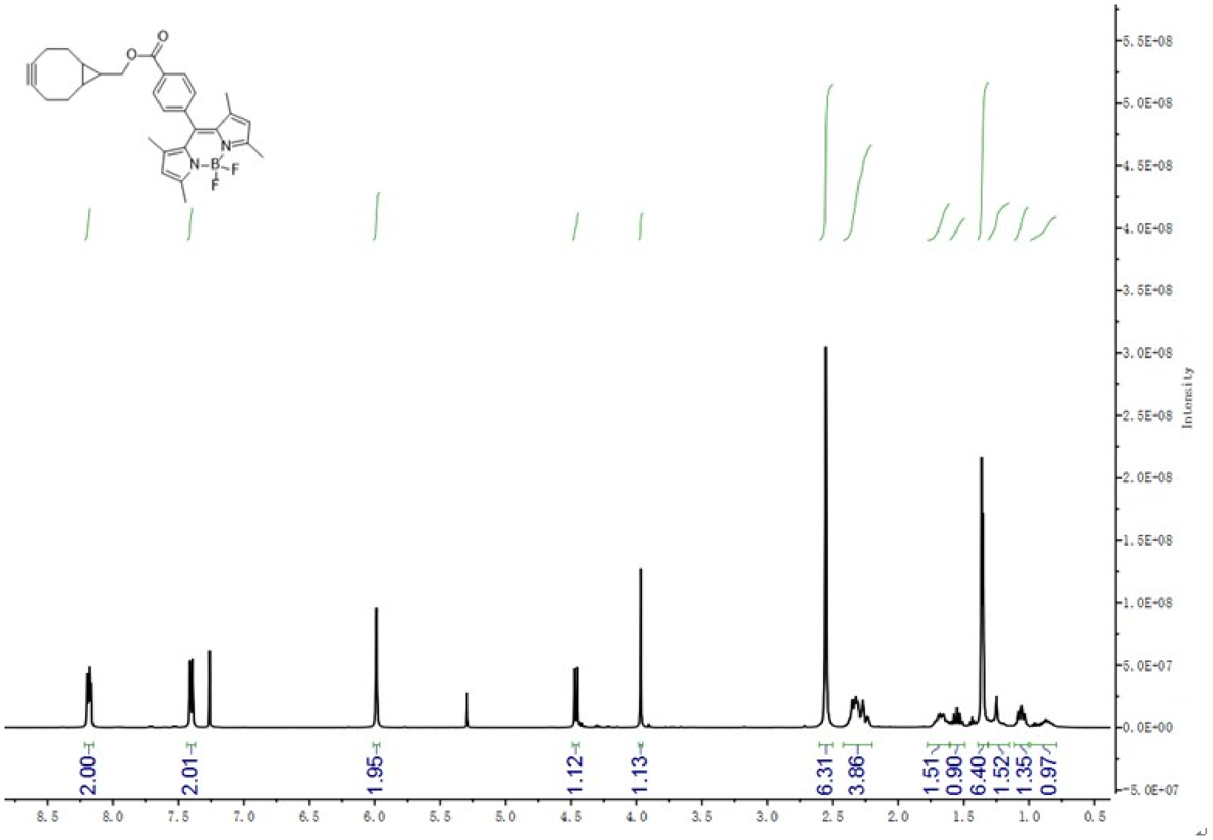

N
B
F
F
2.00
2.01
1.95
1.12
1.13
6.31
3.86
1.51
0.90
6.40
1.52
1.35
0.97
Intensity

^{13}C-NMR

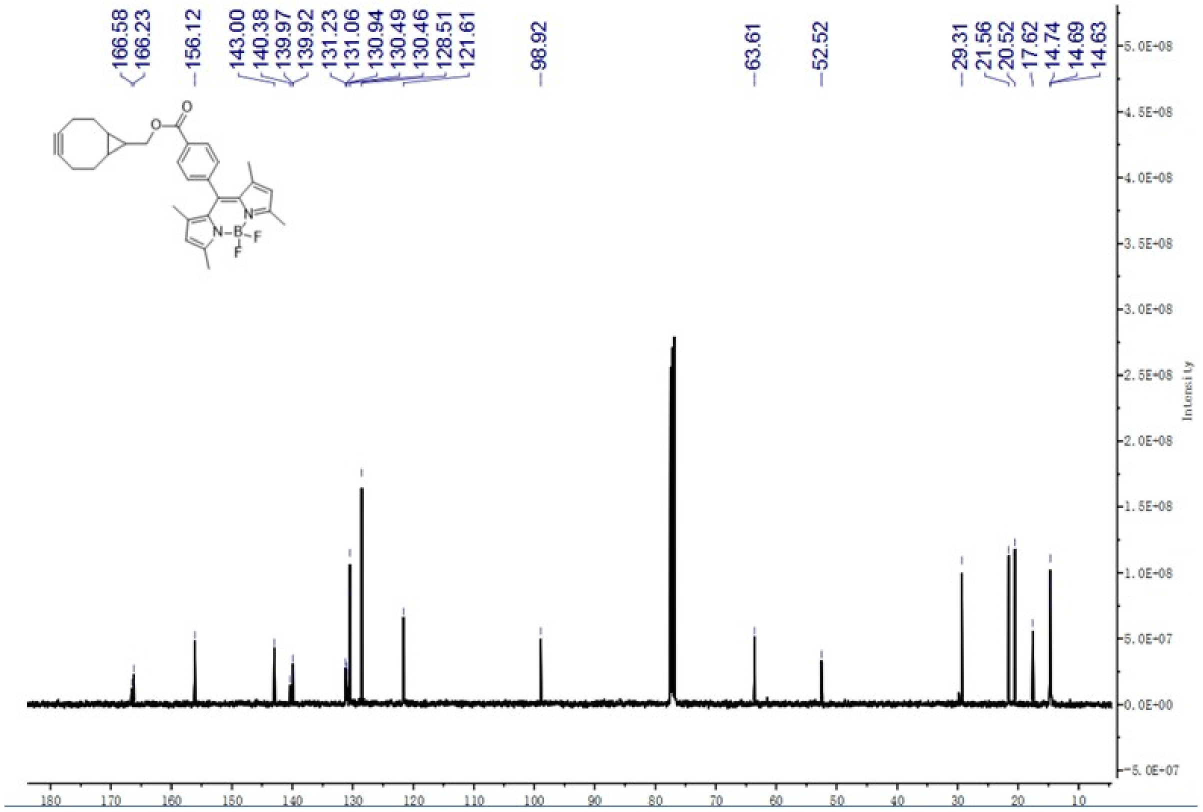

166.58
166.23
156.12
143.00
140.38
139.97
139.92
131.23
131.06
130.94
130.49
130.46
128.51
121.61
98.92
63.61
52.52
29.31
21.56
20.52
17.62
14.74
14.69
14.63
N
B
F
F
Intensity

HRMS

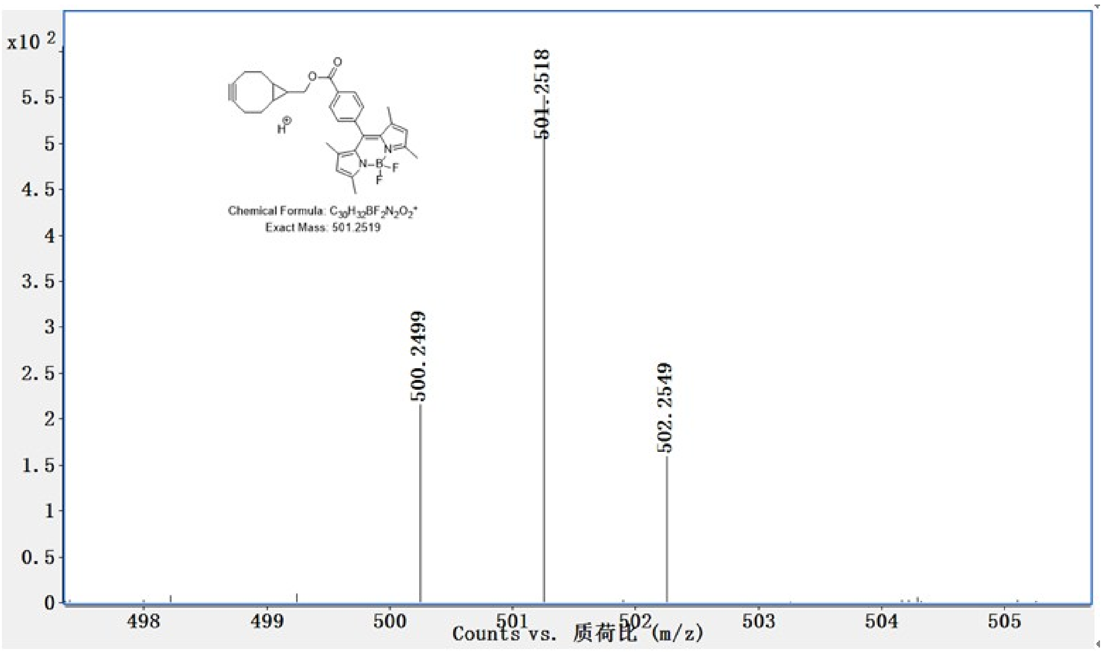

^{1}H-NMR

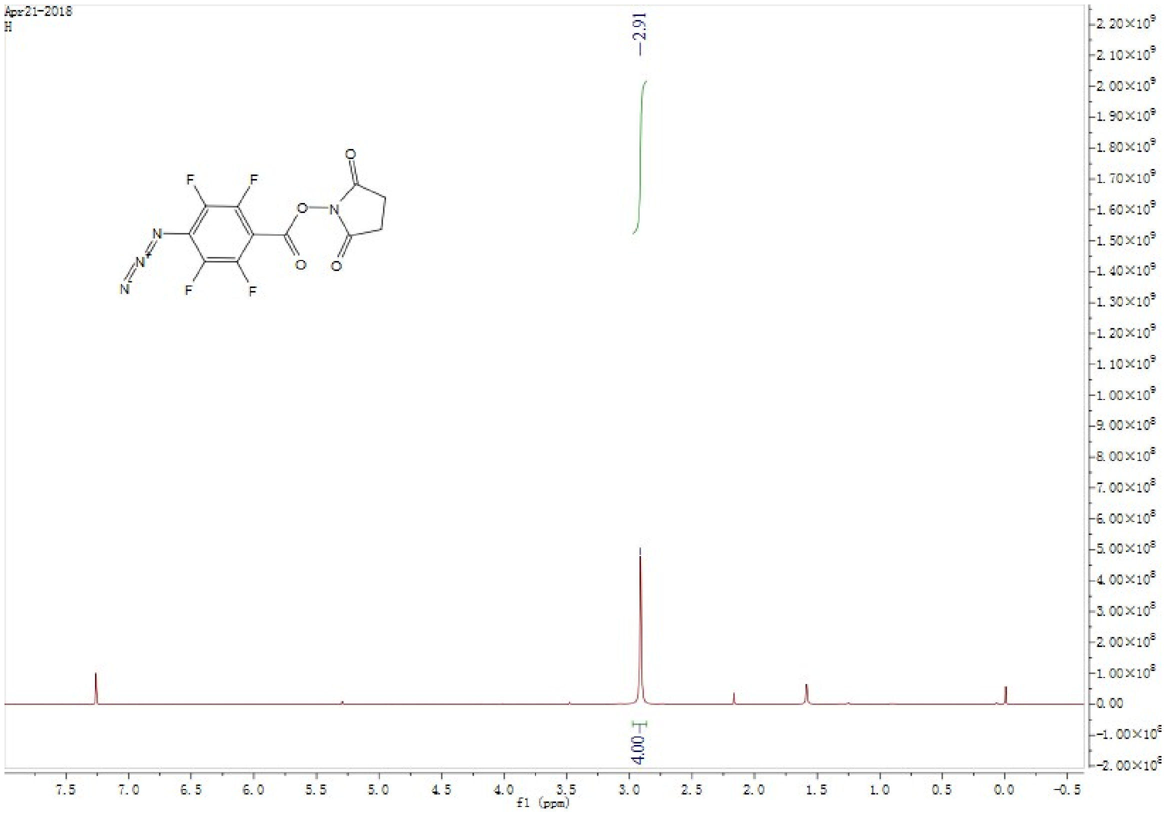

^{13}C-NMR

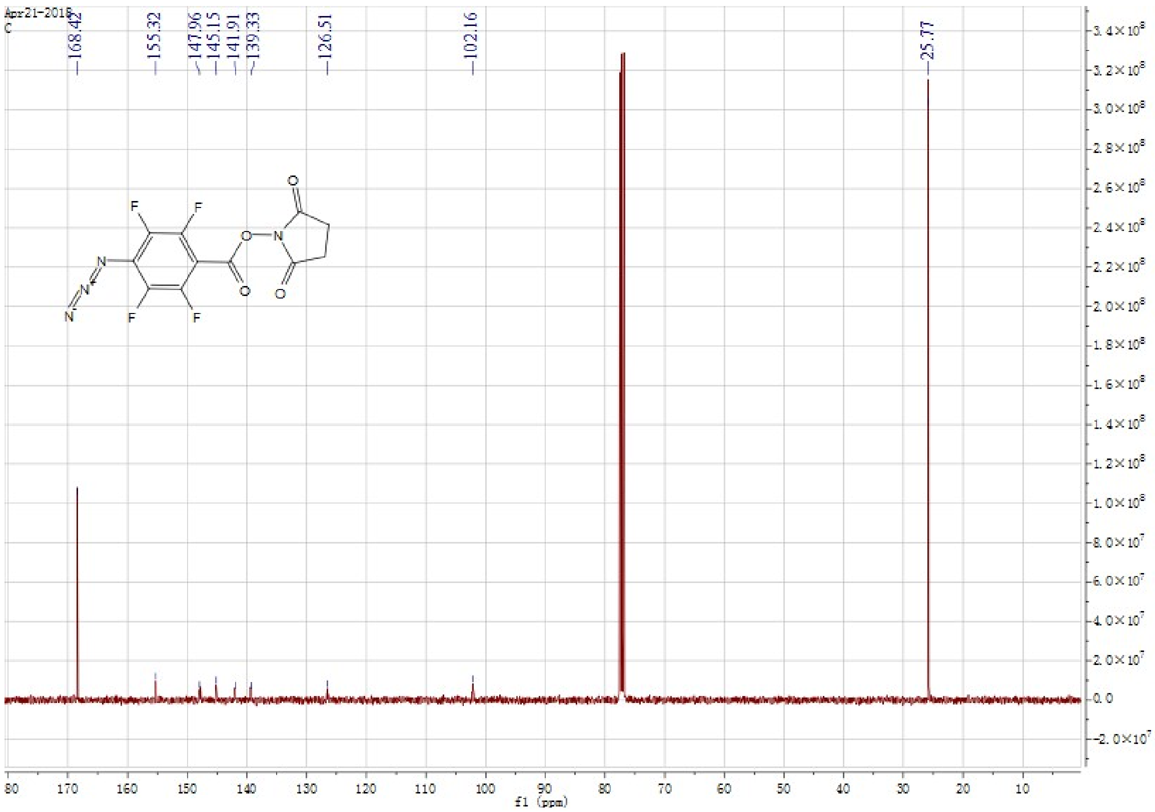

^{19}F-NMR

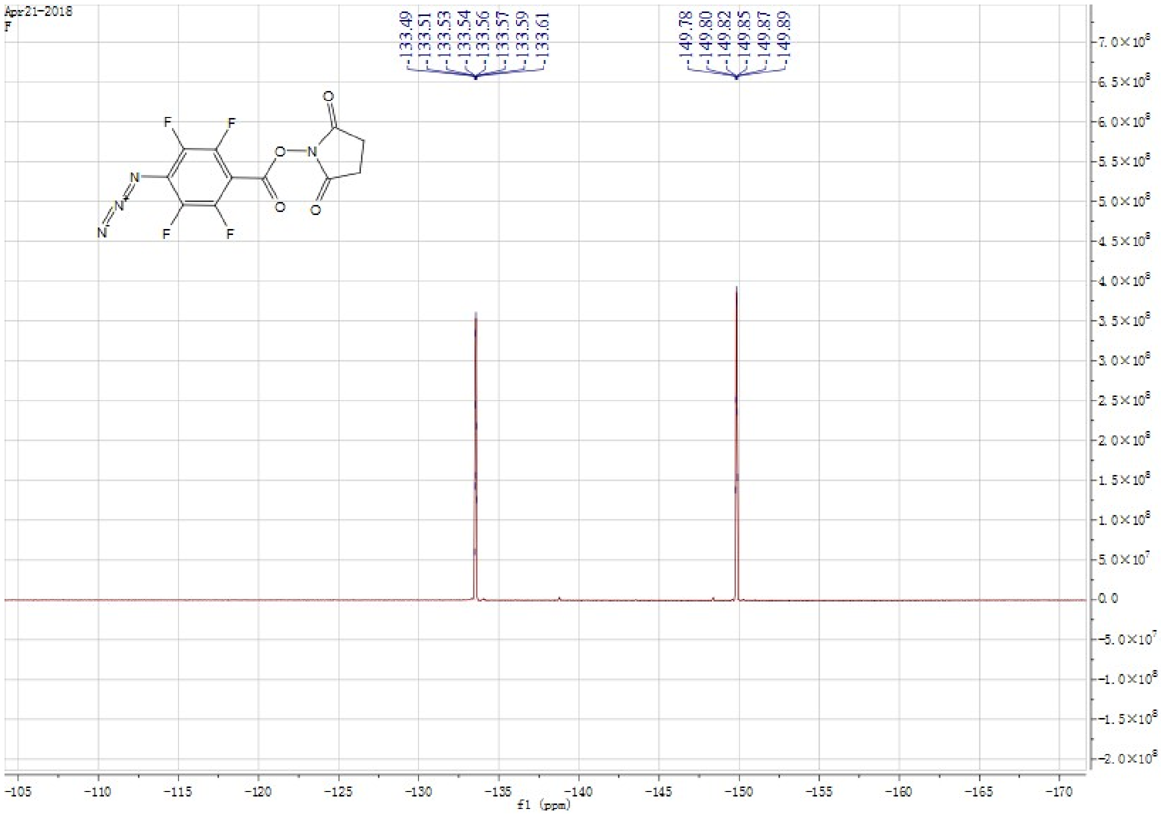

^{1}H-NMR

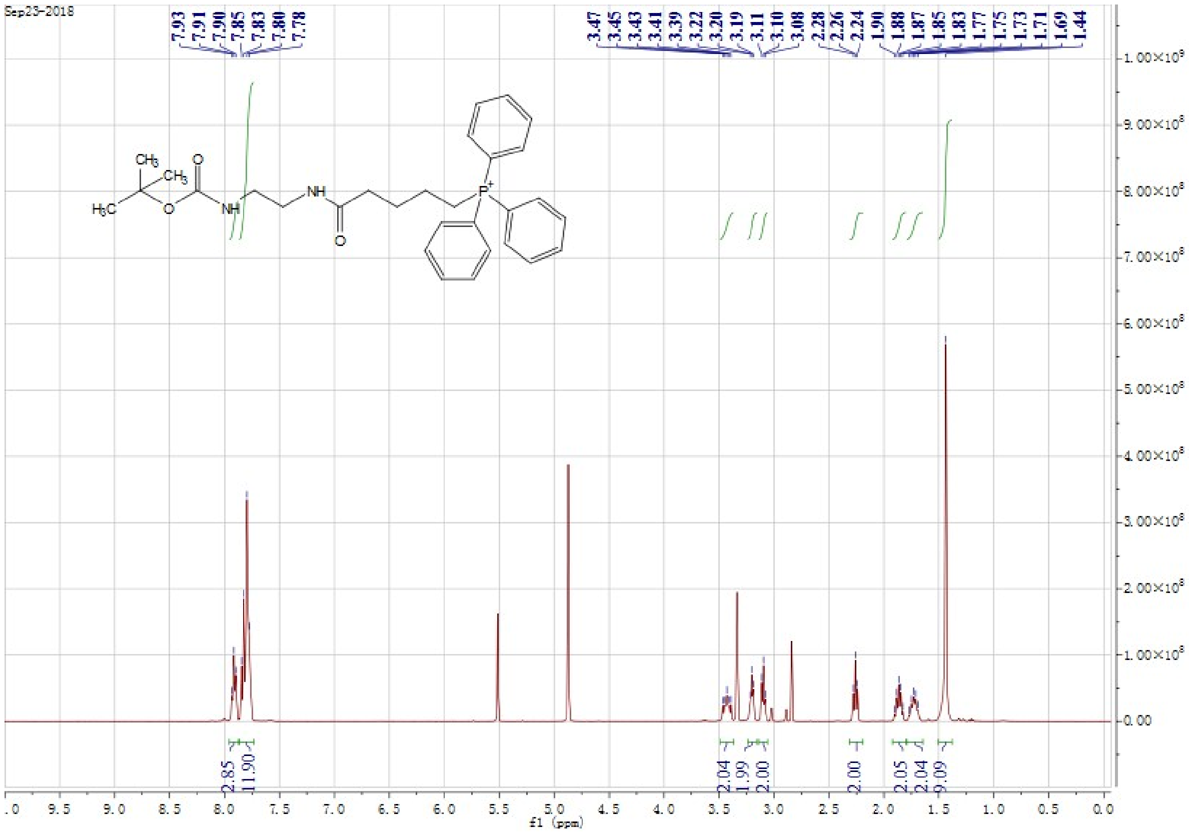

^{1}H-NMR

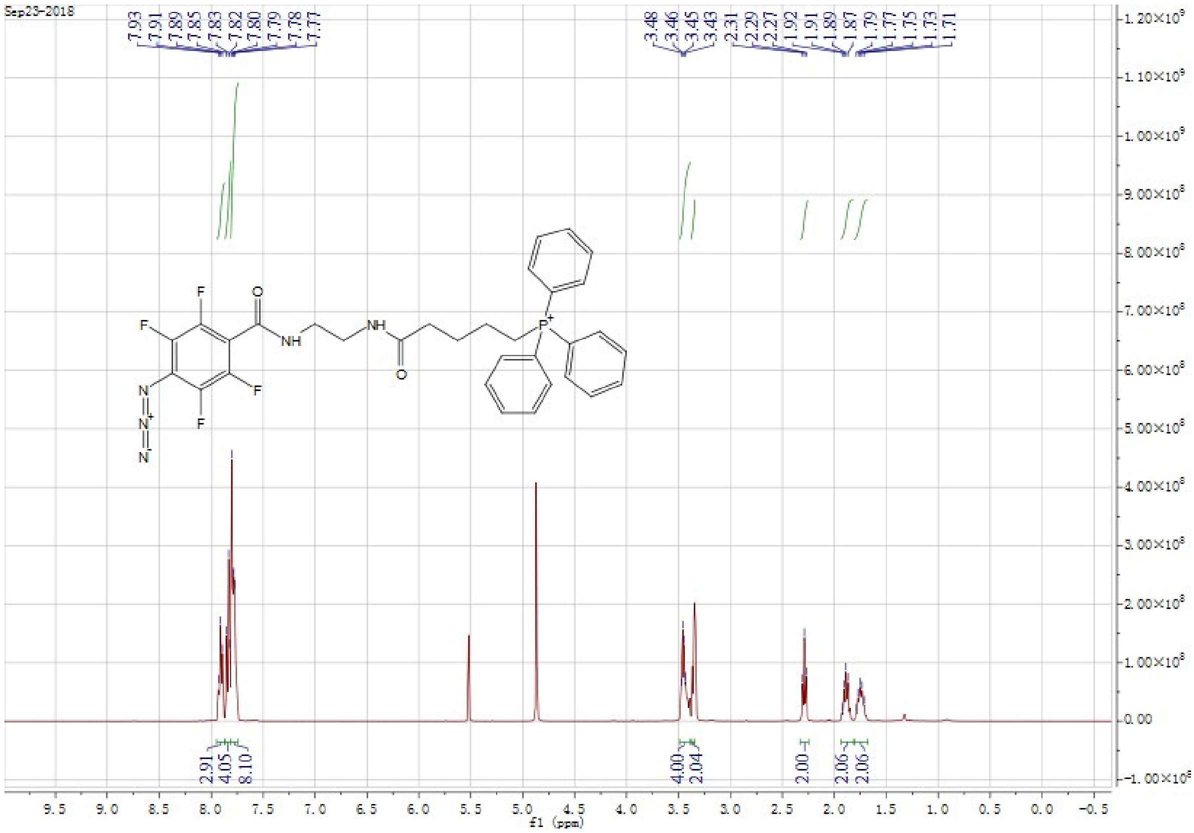

^{13}C-NMR

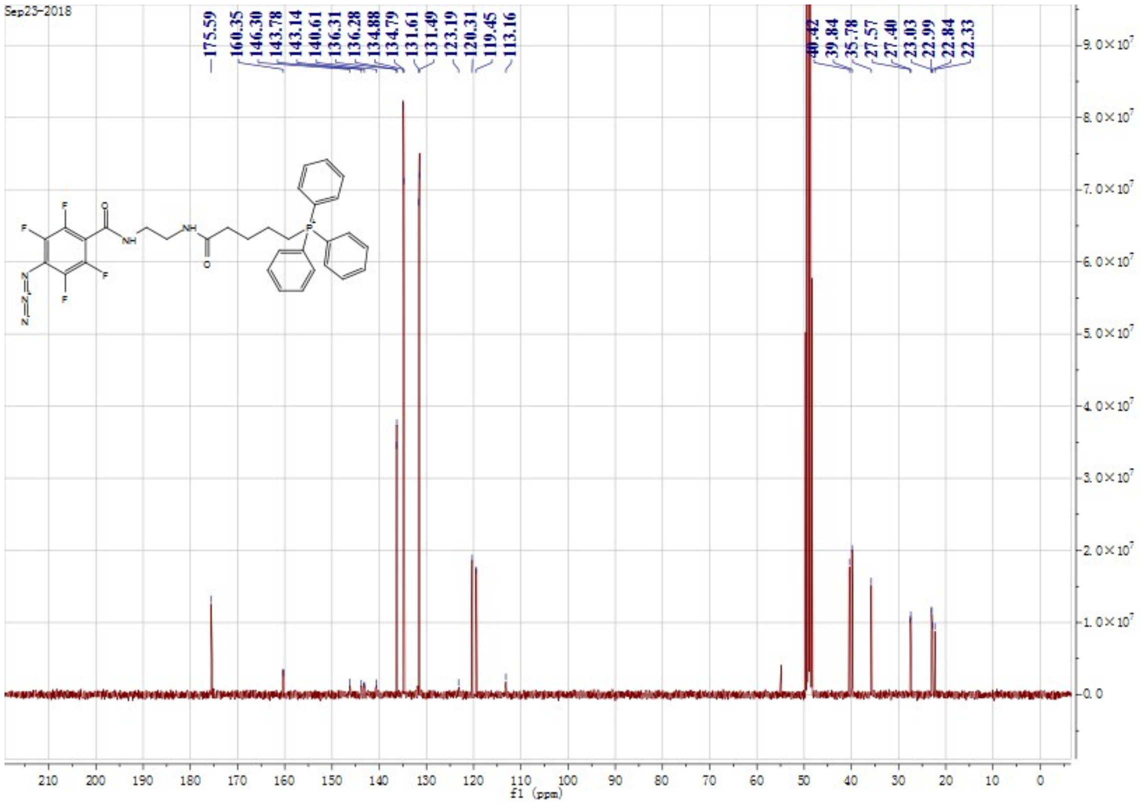

^{19}F-NMR

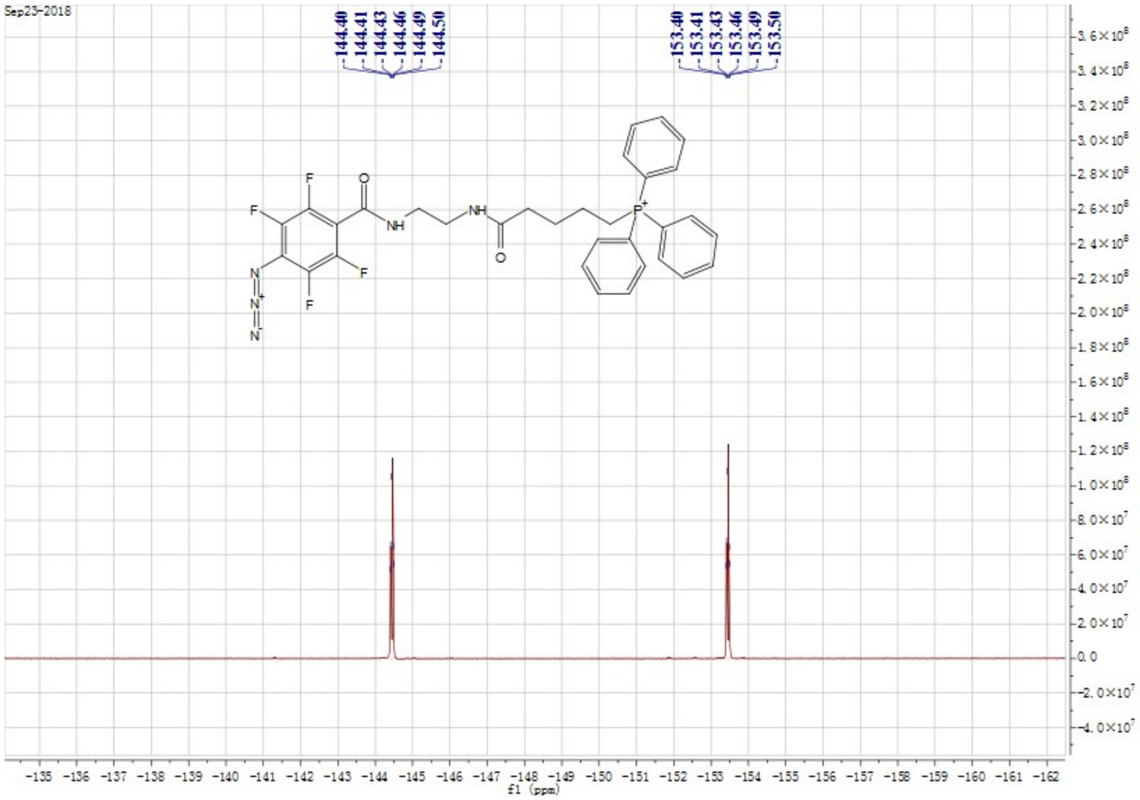

^{31}P-NMR

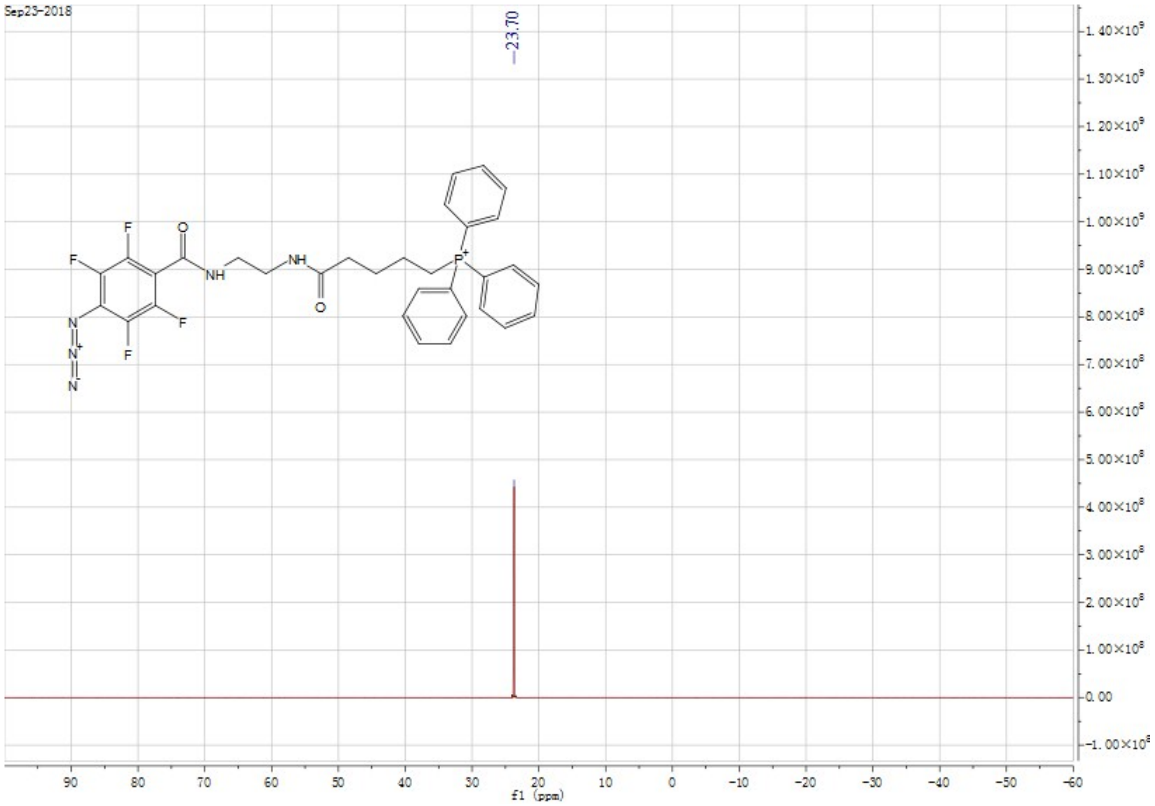

^{13}C-NMR

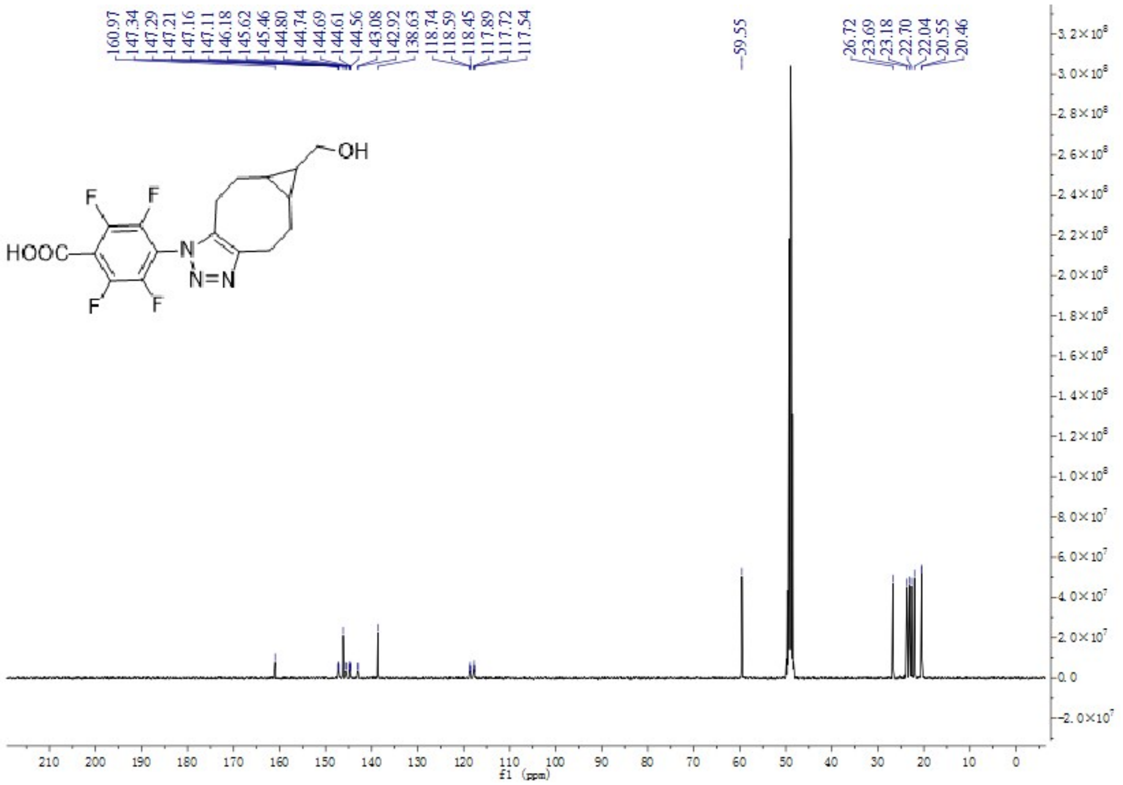

^{19}F-NMR

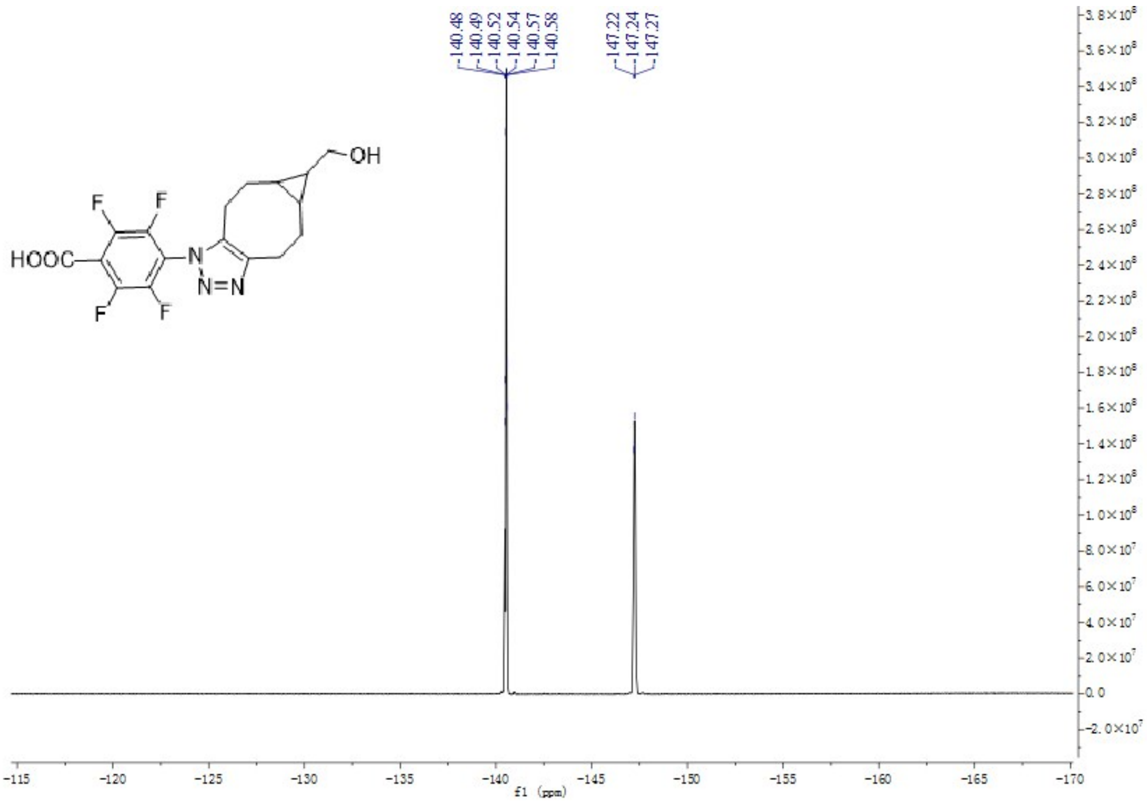
140.48
140.49
140.52
140.54
140.57
140.58
147.22
147.24
147.27
OH
F
F
HOOC
N
N
N
F
F
f1 (ppm)
